# Verbal Memory Impairments in Children after Cerebellar Tumor Resection

**DOI:** 10.3233/BEN-2008-0216

**Published:** 2009-06-01

**Authors:** Matthew P. Kirschen, Mathew S. Davis-Ratner, Marnee W. Milner, S. H. Annabel Chen, Pam Schraedley-Desmond, Paul G. Fisher, John E. Desmond

**Affiliations:** ^1^Department of RadiologyStanford UniversityStanfordCAUSA; ^2^Neurosciences ProgramStanford UniversityStanfordCAUSA; ^3^Department of NeurologyPediatricsNeurosurgery and Human BiologyStanford UniversityStanfordCAUSA; ^4^Department of PsychiatryBrown UniversityProvidence RIUSA; ^5^Department and Graduate Institute of PsychologyNational Taiwan UniversityTaipeiTaiwan; ^6^Division of PsychologyNanyang Technological UniversitySingapore; ^7^Department of NeurologyJohns Hopkins UniversityBaltimoreMDUSA

**Keywords:** Cerebellum, working memory, brain tumor, magnetic resonance imaging

## Abstract

This study was designed to investigate cerebellar lobular contributions to specific cognitive deficits observed after cerebellar tumor resection. Verbal working memory (VWM) tasks were administered to children following surgical resection of cerebellar pilocytic astrocytomas and age-matched controls. Anatomical MRI scans were used to quantify the extent of cerebellar lobular damage from each patient's resection. Patients exhibited significantly reduced digit span for auditory but not visual stimuli, relative to controls, and damage to left hemispheral lobule VIII was significantly correlated with this deficit. Patients also showed reduced effects of articulatory suppression and this was correlated with damage to the vermis and hemispheral lobule IV/V bilaterally. Phonological similarity and recency effects did not differ overall between patients and controls, but outlier patients with abnormal phonological similarity effects to either auditory or visual stimuli were found to have damage to hemispheral lobule VIII/VIIB on the left and right, respectively. We postulate that damage to left hemispheral lobule VIII may interfere with encoding of auditory stimuli into the phonological store. These data corroborate neuroimaging studies showing focal cerebellar activation during VWM paradigms, and thereby allow us to predict with greater accuracy which specific neurocognitive processes will be affected by a cerebellar tumor resection.

## 1. Introduction

As early as 1809, anatomists and physiologists such as Rolando and Flourens concluded, based on ablation experiments in animals, that the cerebellum is "the organ controlling locomotion" [18]. The notion that the cerebellum is primarily or exclusively engaged in activities of motor control and balance has been widely accepted in clinical neurology for decades. Recent findings from neuroimaging and patient studies, however, have suggested that the cerebellum also plays a role in higher-order, non-motor processes such as learning, memory and emotion. A growing body of evidence suggests that children can suffer intellectual disabilities after cerebellar tumor resection [9,13,26,32,34,41, 43,45,53,54,57,64,65]. Due to the heterogeneity of tumor pathology, tumor location and treatment modalities (e.g., irradiation and chemotherapy) used in these studies, it has been difficult to make inferences as to the role of the cerebellum in specific cognitive processes. In addition, most of the studies exploring cognition in cerebellar damaged patients are limited in their abili-

ISSN 0953-4180/08/$17.00 © 2008 – IOS Press and the authors. All rights reserved ty to determine which specific cerebellar regions (i.e. lobules) are responsible for specific cognitive impairments.

Much of our current knowledge of cerebellar function is derived from studies of adult patients with cerebellar pathology. For example, studies of patients with isolated cerebellar infarcts have implicated a role for the right posterolateral cerebellum in verb generation [30], linguistic production [61], and spatial cognition (e.g. hemineglect) [62], whereas the left posterior cerebellum as been associated with the cognitive affective syndrome [50]. In a comparison of patients with isolated posterior inferior cerebellar artery (PICA) and superior cerebellar artery (SCA) territory infarcts, Exner and colleagues showed that patients with inferior and posterior cerebellar damage (including lobules VIIb, VIIIa, VIIIb, IX and Crus II bilaterally) performed worse on tests of verbal episodic long term memory, short term memory and attention than patients with anterior and superior lesions [27]. In a similar study Neau et al. demonstrated no statistical differences between patients with isolated infarcts in the PICA and SCA regions on tests of verbal cognition [46]. However, patients with SCA territory infarcts showed decreased performance on a task of inhibition (i.e. the Stroop naming task with and without interference). Qualitatively, five patients with PICA territory infarcts showed impairments on tests of learning and interference, memory (recall and recognition), and verbal fluency.

The present study was designed to assess the role of specific cerebellar regions for human cognition. Verbal working memory (VWM), the process by which finite units of information are maintained in memory for a brief period of time, served as an assay for cognition in these experiments. Baddeley proposed a framework for VWM, called the phonological loop, which consists of two sub-components, a phonological short-term store, which can hold speech-related information for 1–2 seconds, and an articulatory control system, which serves to sub-vocally refresh the contents of the phonological store [4,5,8]. Using functional magnetic resonance imaging (fMRI) activations elicited from a VWM task and known cerebro- cerebellar projections [56], Desmond and colleagues proposed an extension to Baddeley's VWM framework, in which both the superior and inferior cerebellar hemispheres provide supportive processing to enhance efficiency of neocortical functions through a feed-forward network [23].

It is well known that VWM continues to develop throughout childhood and adolescence [28, 29]. Although the majority of studies investigating VWM through psychophysical tasks and neuroimaging paradigms have focused on adult populations, a few studies have examined VWM and its neural correlates during various stages of development [11,12,35,48]. In one of the earliest studies of functional neuroimaging with children, Casey showed activation of inferior and middle frontal gyri during performance of a VWM task which correlated to areas visualized in adults by Cohen et al. [19]. Diffusion tensor and functional MR imaging results from another study of children demonstrated activation of superior frontal and inferior parietal regions [48], both classic centers underlying VWM processing in adults. Brahmbhatt and colleagues [11] further demonstrated activation of the known functional network subserving VWM, including the right cerebellum, and found that certain regions within these networks, specifically the superior parietal cortex, show age-related differences between adolescents and adults. Similarities in functional neuroanatomy between adults and children have also been demonstrated in several studies for spatial working memory [39,40,47,67].

Currently, patients with cerebellar pathology are not routinely evaluated for cognitive deficits. Even if they are administered a standard neuropsychological assessment, comprehensive tests of phonological storage and articulatory rehearsal, the two sub-components of VWM, are not typically included. Therefore, to assess for the necessity of the cerebellum's contribution to the phonological loop, we applied a series of computerized psychophysical tasks to systematically probe the two sub-components of VWM. In addition, detailed anatomical analyses were performed to determine precisely which cerebellar lobules were compromised for each patient. Based on patterns of fMRI activation during verbal working memory [23], we hypothesized that articulatory rehearsal deficits would be correlated with damage to superior cerebellar hemispheres and portions of the vermis. Deficits in phonological storage, on the other hand, should be correlated with damage to inferior cerebellum. It is conceivable that if the cerebellum does play an important role in the process of VWM, many of the higher-order cognitive deficits described in cerebellar damaged patients could potentially be explained by this involvement.

## 2. Subjects and methods

### 2.1. Subjects

Subjects were 12 patients recruited through the Pediatric Neuro-Oncology Clinic at Lucile Packard Chil-

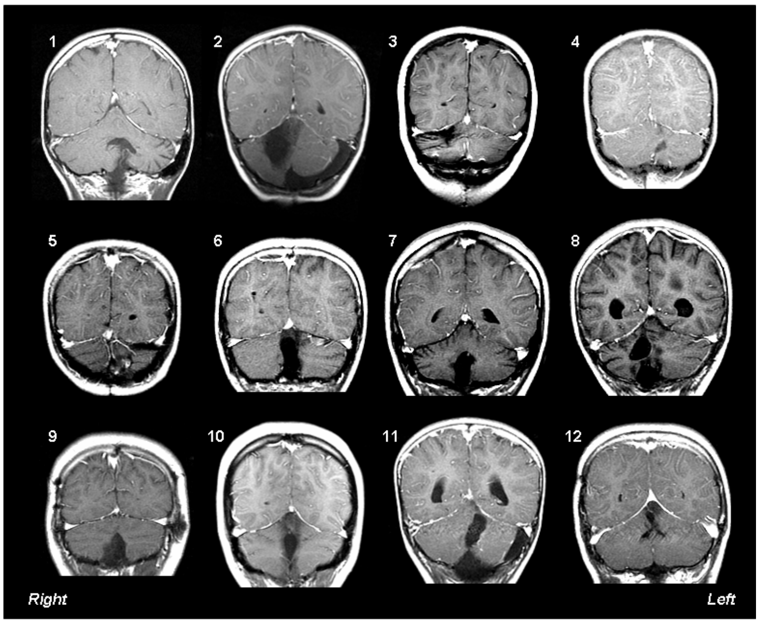

Fig. 1. T1-weighted coronal MRI images showing the cerebellum of all 12 patients after tumor resection. Image numbers correspond to subject numbers in Table 2. Images are presented in radiological convention.

dren's Hospital who had undergone complete surgical resection for a cerebellar pilocytic astrocytoma, without any adjuvant radiotherapy or chemotherapy, and an equal number of gender and age-matched healthy controls. Representative coronal sections illustrating the damage to the cerebellum are depicted in Fig. 1. Patients ranged from 6–19 years old (mean = 12.5 years, S.D. = 4.1, distribution = 13.9, 6.5, 8.4, 8.5, 11.6, 14.0, 19.4, 15.0, 14.9, 17.9, 8.4, 11.4 years) and were an average of 5.5 $\pm$ 3.1 (SD) years post surgical resection. Control subjects were selected to match age and education level of the patients, and statistical tests confirmed the lack of any significant differences in either age ($t(11) = 0.31$, $p = 0.764$) or level of education ($t(11) = -0.56$, $p = 0.586$) between patients and controls. Informed consent was obtained from each individual subject prior to their participation, which was approved by the Institutional Review Board at Stanford University. All subjects received limited financial compensation for their participation.

All subjects spoke English fluently and all but one patient were right handed. All patients had a normal neurological exam without any gross cerebellar deficits at the time of testing and none reported a history of head injury with loss of consciousness or diagnosis of a psychological disorder before or after their tumor resection. In addition, none of the patients or controls reported being diagnosed with a learning disability or an attention deficit disorder, or being enrolled in special education courses. All healthy controls also reported no history of a head injury with loss of consciousness, neurological or psychological disorders, or major illnesses or surgeries.

### *2.2. Neuropsychological assessment*

Subjects completed the following battery of neuropsychological tests to assess general intellectual functioning, specific components of the VWM circuitry (e.g. phonological processing), and fine motor coordination:

*Wechsler Abbreviated Scale of Intelligence* (*WASI*$^{TM}$) [75]. The WASI has been shown to be a reliable measure of intelligence. The two subtests, vocabulary and matrix reasoning, were used.

*Conner's Continuous Performance Test-Second Edition* (*CPT-II*) [20]. The CPT-II measures attention and simple reaction time.

*Comprehensive Test of Phonological Processing* (*CTOPP*) [73]. The CTOPP measures phonological processing abilities.

*Controlled Oral Word Association* (*COWA*) [63]. The COWA test measures both phonemic and semantic verbal fluency.

*Purdue Pegboard Test* [68]. The Purdue Pegboard measures finger and hand dexterity for fine motor coordination. This test has been used to localize cerebral lesions and deficits [52].

### 2.3. Behavioral VWM tasks

The most fundamental VWM task requires subjects to recall serial sequences of digits of increasing length. Pre-recorded sequences of one, two, three, four, five, six or seven digits (0–9) were presented either aurally through audio speakers or visually on a computer monitor. Subjects received 5 trials at each sequence length, beginning with one item. If a subject failed to correctly recall at least 2 sequences at a given length the following longer sequence was not administered. Memory span for each condition was calculated by summing the accuracy for each of the seven sequence lengths [60, 71]. Memory span was measured using two modes of response. For the first, subjects verbally repeated the sequence; for the second, a $2 \times 5$ array of letters, randomly arranged, were presented on the computer screen and subjects pointed to the items in sequence.

The immediate serial recall of phonologically dissimilar items is generally more accurate than recall for phonologically similar items, a phenomenon that has been referred to as the phonological similarity effect (PSE) [7,21]. This phenomenon is thought to arise because of conflicts in the phonological store. Thus, impairments in the phonological store should result in an attenuation of the PSE and similar performance for both phonologically similar and dissimilar stimuli (i.e. a negative PSE). Subjects were instructed to remember a list of six consonants taken from a set of either phonologically similar (B, C, D, G, P, T, V) or phonologically dissimilar (F, K, Q, R, X, W, Z) letters presented at a rate of 2 items per second either aurally or visually through a computer system. After subjects rehearsed these letters sub-vocally during a 5000 ms retention interval, they responded "yes" or "no" via a button press to indicate if a probe item, presented for the initial 1500 ms of a 2000 ms response interval, matched a remembered letter in the preceding list. The probe and response period was followed by an inter-trial-interval of 3000 ms. A fixation cross, presented for 750 ms (followed by a 250 ms delay) indicated the start of each trial. Subjects completed 48 trials of the PSE task in each presentation modality.

The continuous uttering of irrelevant speech (e.g., repeating the words "the" or "blah") during a VWM task disrupts the articulatory rehearsal system and leads to impairments in performance [6]. To test this articulatory suppression effect, subjects were instructed to remember a list of six randomly generated consonant letters presented at a rate of 2 per second either aurally or visually. During half of the trials, subjects were instructed to start uttering the syllable "blah" before the presentation of the stimuli, and to stop after the 5000 ms sub-vocal rehearsal period when the probe item was presented. Subjects again responded via a button press to the probe and completed 48 trials of the task in both visual and auditory modalities. The inter-trial-interval for this task was 3000 ms, and trials also began with a 750 ms fixation cross followed by a 250 ms delay.

Another way to assess the integrity of VWM circuitry is to measure subjects' performance when they freely recall sequences of stimuli that exceed the length of their immediate memory span. Normal subjects show greater recall of the final items in the list (recency effect), compared to the preceding stimuli [31,71,72]. In immediate auditory free recall, the phonological short term store provides a greater contribution to the recency effect than the articulatory control system [72, 74]. Thus, patients with an impaired phonological store should show an attenuation of the recency effect. On the other hand, patients with a reduced memory span and a normal recency effect, the deficit could be reasonably attributed to the articulatory rehearsal process [71].

Twenty lists, composed of 12 common nouns, chosen at random and without replacement from a pool of high frequency nouns [58], were visually presented to the subjects at a rate of 1 item per second. Immediately after the presentation of the list, subjects were instructed to verbally recall as many words as they could remember in any order. The words were manually recorded by the experimenter in the order they were recalled (an audio tape recording was used to verify recalled words). Subjects indicated they were ready to proceed to the next list by pushing a button. A cross, presented for 1 second, indicated the start of a new trial.

### 2.4. Anatomical analysis of cerebellar tumors

In order to determine the effect of a particular cerebellar resection on behavioral performance, coronal T1-weighted multi-echo multi-planar post-surgical MRI brain images were acquired for each subject from clinical scans. These scans consisted of 24–30 coronal sections 5 mm thick with 1 mm gap and an inplane resolution of 0.43 mm–0.94 mm. Repetition time (TR) ranged from 417–800 ms, and echo time (TE) was 20 ms. These slices were assembled into a 3D volumetric data set [66] and aligned into the Montreal Neurological Institute (MNI) coordinate system. Using custom-made

Table 1
Neuropsychological assessment scores

| Neuropsychological[1] measure | Controls (Mean ± SD) | Patients (Mean ± SD) | Significance |
|---|---|---|---|
| WASI | | | |
| FSIQ[2] | 119.5 ± 15.9 | 105.7 ± 13.0 | $p = 0.029^*$ |
| Vocab | 63.6 ± 16.2 | 57.0 ± 8.5 | $p = 0.225$ |
| Matrix | 57.3 ± 6.8 | 49.0 ± 10.7 | $p = 0.032^*$ |
| COWA | | | |
| FAS | 53.7 ± 15.2 | 51.8 ± 14.5 | $p = 0.755$ |
| Animal Naming | 54.3 ± 15.1 | 50.9 ± 12.6 | $p = 0.554$ |
| CTP-II[3] | 53.2 ± 9.6 | 52.4 ± 12.0 | $p = 0.858$ |
| CTOPP[2] | | | |
| PAQ | 115.7 ± 8.8 | 111.2 ± 8.9 | $p = 0.225$ |
| PMQ | 109.0 ± 15.9 | 99.8 ± 12.7 | $p = 0.129$ |
| RNQ | 105.8 ± 14.6 | 101.5 ± 13.1 | $p = 0.461$ |
| Purdue Pegboard | | | |
| Preferred hand | 48.8 ± 11.1 | 42.1 ± 13.4 | $p = 0.199$ |
| Non-preferred hand | 39.0 ± 12.2 | 37.4 ± 17.0 | $p = 0.795$ |
| Both hands | 39.0 ± 5.8 | 32.7 ± 13.2 | $p = 0.144$ |

[1] All measures are represented in T scores (mean = 50 and SD = 10) unless otherwise stated.
[2] Standard scores (mean = 100 and SD = 15).
[3] Only 10 out of 12 patients performed this test.
[*] Significant at $p < 0.05$.

software previously described [24], this brain volume was then realigned such that the brainstem was oriented vertically as visualized on a midline sagittal image. The x, y, and z extent of the cerebellum in this new orientation was measured manually for each patient using a utility built in to the software, thereby allowing the volume to be adjusted for potential age-related differences in cerebellar dimensions and scaled to match the cerebellar dimensions of the MNI template. The net result was a 9-paramter (translation, rotation, and scaling adjustments in the x, y, z dimensions) normalization for each subject to the MNI cerebellum. Eleven coronal sections were then resliced parallel to the brainstem, and the perimeter of the surgical resection was manually outlined from these sections using a closed polygon utility that captured the x, y, z coordinates of all the voxels contained within the polygon. From these captured coordinates a lesion "volume of interest" could be created in the same volume coordinate system. The lobular distribution of each lesion was determined by overlaying the lesion volume of interest on a separate volume containing anatomical codes from the Automated Anatomical Labeling (AAL) map [69] distinguishing the individual cerebellar lobules. Deep (dentate/interposed) nucleus regions of interest were created using probabilistic X, Y, Z dimensional boundaries for the MNI brain published by Dimitrova et al. [25]. Values corresponding to 51–60% of subject overlap were used (see their Table 1) in the ROI creation. The percentage of voxels occupied by the lesion in each lobule or deep nuclear ROI was computed and recorded.

All patients were confirmed to have isolated pilocytic cerebellar astrocytomas. A complete set of neuroimaging scans, including T2-weighted scans and contrast studies, were inspected to ascertain that no other tumors or lesions were present.

### 2.5. Statistical analyses

#### 2.5.1. Analysis of main behavioral variables

The effects of cerebellar damage on verbal digit span were assessed with a 2 × 2 repeated measures analysis of variance (ANOVA) with one between subject factor of group (patient vs. control) and one within subject factor of modality of presentation (auditory vs. visual). The effects of cerebellar damage on articulatory control were assessed by comparing performance (i.e., accuracy, defined as the proportion of the total number of trials in which a correct response was made) with and without articulatory suppression, and was evaluated using a 2 × 2 × 2 repeated measures ANOVA with within subject factors of modality (auditory vs. visual) and rehearsal type (with vs. without suppression), and between subject factor of group. The effects of cerebellar damage on phonological storage were assessed with two analyses. First, the phonological similarity effect was evaluated by comparing performance (i.e., accuracy, as defined above for articulatory suppression) with phonologically similar vs. dissimilar letters. A $2 \times 2 \times 2$ repeated measures ANOVA was therefore used for analysis with within subject factors of modality (auditory vs. visual) and phonological similarity (similar vs. dissimilar), and between subject factor of group. Second, the recency effect of freely recalled items was assessed by examining accuracy of recall as a function of serial position of presentation. Statistical analysis of this effect was conducted using a $2 \times 12$ repeated measures ANOVA with within subject factor of serial position and between subject factor of group (this test was conducted only with visual presentation so there is no factor of modality). Because two neuropsychological test measures (FSIQ and Matrix measures, see below) were significantly different between the groups, ANOVAs that indicated significant group effects were repeated using each of these measures as a covariate to insure that significant group effects of interest remained significant.

#### *2.5.2. Mapping behavioral performance to lesion locations*

For behavioral measures in which patient performance was found to be significantly worse than that of controls, regression analyses were conducted to determine if the extent of lobular damage was correlated with the degree of behavioral impairment. If the behavioral measure was found to be correlated with the age of the patient, both lobular damage and age were regressed on the behavioral measure and partial correlations were computed to determine if lobular damage significantly predicted performance above and beyond the contribution of age. To correct for multiple comparisons (24 lobular/deep nuclear regions of interest) a Bonferroni correction was applied to achieve an overall family wise error rate of 0.05, taking into account for the fact that cerebellar region of interest measures were correlated with each other [55]. The mean correlation of the regions of interest was determined by averaging all 276 pairwise combinations of correlations from the 24 regions of interest, and this average was 0.325. From these values, a Bonferroni corrected p value of 0.00585 was required.

## 3. Results

### *3.1. Neuropsychological measures*

Table 1 displays results of the neuropsychological assessment measures from patients and their age-matched controls. Results from the WASI (vocabulary and matrix subtests), COWA, CPT-II and Purdue Pegboard are presented as T-scores with a mean of 50 and standard deviation of 10. Results from the WASI (full scale IQ) and CTOPP are presented as standard scores with a mean of 100 and a standard deviation of 15. Although all of the tumor patients except for one had IQ scores in the average or above average range, overall, the patients scored significantly lower than their respective controls (t(22) $= -2.336$, $p = 0.029$). This effect was carried primarily by the matrix subtest of the WASI which was significantly higher for controls (t(11) $= -2.29$, $p = 0.032$). There was no significant difference between patients and controls on tests of verbal fluency (FAS), animal naming, attention (CPT-II), phonological processing (CTOPP), or fine motor control (pegboard, preferred hand).

As described above, one cerebellar patient was left handed. The specific neuropsychological scores for this patient were as follows: WASI: FSIQ = 95, Vocab = 59, Matrix = 35; COWA: FAS = 49, Animal Naming = 56; CTP-II = 61.4; CTOPP: PAQ = 112, PMQ = 127, RNQ = 85; Pegboard: Preferred Hand = 43.7, Non-Preferred Hand = 20.5, Both Hands = 16.2. For the behavioral tests described above, the main abnormality displayed for this patient was an abnormal phonological similarity effect to visually presented stimuli. The lesion for this subject was located in the vermis, right inferior cerebellum, and right deep nuclei.

### *3.2. Anatomical analyses*

Figure 2 illustrates the anatomical distribution of cerebellar damage averaged over the 12 patients. The X axis depicts the names of the lobules and the Y axis depicts the mean percent damage to the lobule. Table 2 summarizes the subject by subject distribution of lobular damage. These analyses indicate that the vermis suffered the greatest amount of damage in these patients.

### *3.3. Behavioral VWM tasks*

Analysis of variance of verbal digit span revealed a significant group x modality interaction (F(1,22) $= 7.69$, $p = 0.011$, Fig. 3), which remained significant when FSIQ and Matrix scores were entered as covariates (group x modality interaction $p = 0.007$ and $p = 0.001$, respectively). Analysis of simple main effects indicated that patients performed worse than controls in the auditory modality (t(11) $= 2.65$, $p = 0.023$, paired

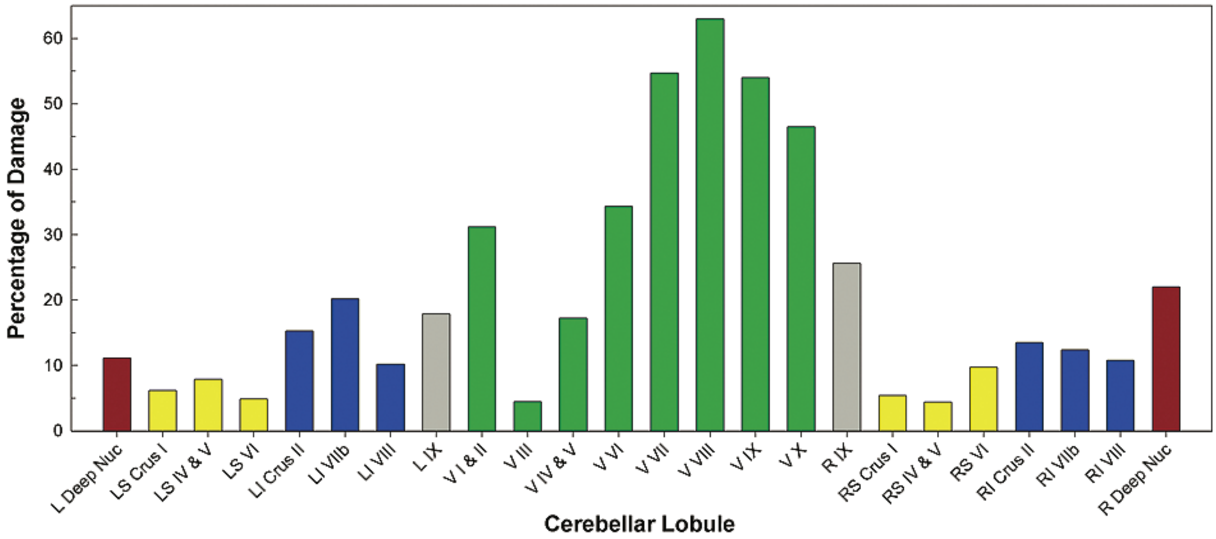

Fig. 2. Anatomical distribution of cerebellar damage averaged over the 12 patients. Mean percent damage to the lobule is plotted against the cerebellar lobules. Yellow bars = superior cerebellar regions; Blue = inferior cerebellar regions; Gray = cerebellar tonsils; Green = cerebellar vermis, Maroon = deep cerebellar nuclei.

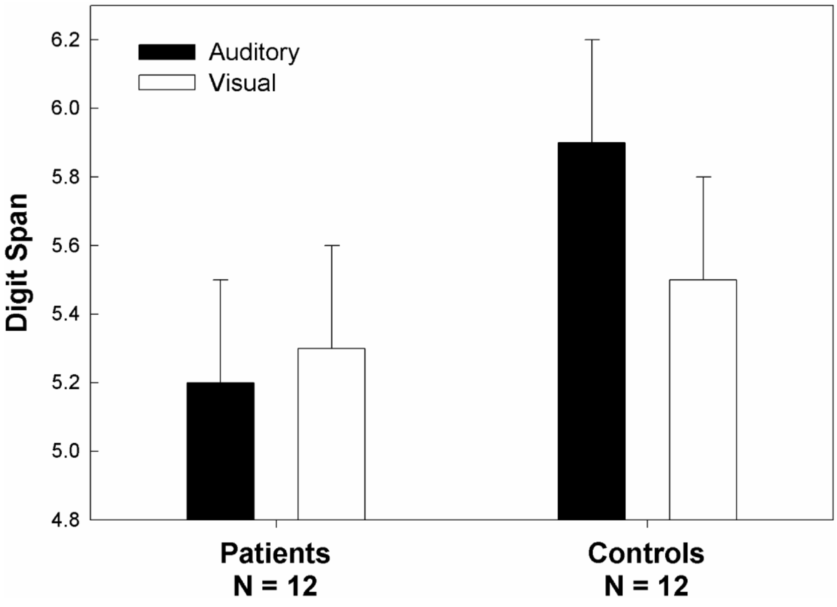

Fig. 3. Digit span performance for patients and controls in auditory and visual modalities. Average digit span performance for 12 patients and 12 controls is depicted and error bars represent standard error of the mean.

t-test of patients with age-matched controls), but not in the visual modality. Analysis of performance when patients pointed rather than verbally responded indicated no difference in the two modes of responding. Patients did not perform significantly different for auditory and visual modalities ($t(11) = 0.532$, $p = 0.605$). Controls on the other hand, performed significantly better in the auditory modality ($t(11) = 4.29$, $p < 0.01$). In addition, the ANOVA indicated a significant main effect of group ($F(1,22) = 5.65$, $p = 0.027$).

Analyses of structure/behavior correlation were confined to the auditory modality because patient and control digit span differed only in this modality. Auditory digit span performance was found to be correlated

Table 2
Distribution of cerebellar lobular damage

| Subject | Left Deep Nuc | Left Superior Crus I | Left Superior IV & V | Left Superior VI | Left Inferior Crus II | Left Inferior VIIb | Left Inferior VIII | IX |
|---|---|---|---|---|---|---|---|---|
| 1 | + | − | − | − | − | + | + | − |
| 2 | − | + | + | + | + | + | + | − |
| 3 | − | − | − | − | − | − | − | − |
| 4 | − | − | − | − | − | − | − | + |
| 5 | − | + | − | − | + | + | − | − |
| 6 | − | − | − | − | + | + | + | + |
| 7 | − | − | − | − | − | + | − | + |
| 8 | ++ | − | + | + | + | + | + | + |
| 9 | − | − | − | − | + | + | + | + |
| 10 | − | − | + | + | + | − | − | + |
| 11 | + | + | + | + | ++ | ++ | + | + |
| 12 | − | − | + | − | − | − | − | − |

| Subject | Vermis I & II | Vermis III | Vermis IV & V | Vermis VI | Vermis VII | Vermis VIII | Vermis IX | Vermis X |
|---|---|---|---|---|---|---|---|---|
| 1 | − | − | − | − | − | − | − | − |
| 2 | − | − | + | ++ | ++ | ++ | + | − |
| 3 | − | − | − | + | + | − | − | − |
| 4 | − | − | − | − | − | + | + | − |
| 5 | − | − | − | − | + | − | − | − |
| 6 | ++ | + | + | ++ | ++ | ++ | ++ | ++ |
| 7 | + | − | − | + | + | ++ | ++ | ++ |
| 8 | ++ | + | + | ++ | ++ | ++ | ++ | ++ |
| 9 | ++ | + | − | + | ++ | ++ | ++ | ++ |
| 10 | + | − | ++ | ++ | ++ | ++ | ++ | ++ |
| 11 | ++ | + | + | + | ++ | ++ | ++ | ++ |
| 12 | − | − | ++ | ++ | ++ | ++ | + | − |

| Subject | IX | Right Superior Crus I | Right Superior IV & V | Right Superior VI | Right Inferior Crus II | Right Inferior VIIb | Right Inferior VIII | Right Deep Nuc |
|---|---|---|---|---|---|---|---|---|
| 1 | − | − | − | − | − | − | − | − |
| 2 | + | + | + | + | + | + | + | ++ |
| 3 | − | + | − | + | + | + | − | − |
| 4 | − | − | − | − | − | − | − | − |
| 5 | − | − | − | − | − | − | − | − |
| 6 | + | − | − | + | + | + | + | − |
| 7 | ++ | − | − | − | + | + | + | + |
| 8 | + | + | + | + | + | + | + | ++ |
| 9 | ++ | − | − | − | + | + | + | + |
| 10 | + | − | + | + | + | + | + | + |
| 11 | + | + | − | − | + | + | + | + |
| 12 | − | − | − | − | − | − | − | − |

−less than 5% of lobule resected; +5–50% of lobule resected; ++more than 50 % of lobule resected.

with patient age (Pearson $r = 0.88$, $p < 0.0001$), so both lobular damage and age were regressed on patient digit span performance, and partial correlations were performed to determine if lobular damage significantly increased the proportion of accountable variance. This analysis resulted in only one significant lobule, the left hemispheral lobule VIII (lobule beta test: t(9) = 3.65, $p = 0.0054$; multiple-R for lobule and age = 0.955, F(2,9) = 46.73, $p < 0.00001$).

Analysis of articulatory suppression revealed a significant rehearsal type x group interaction, indicating that patients were significantly less affected by articulatory suppression than controls (F(1,22) = 5.65, $p = 0.027$, Fig. 4A). This effect remained significant when the ANOVA was repeated with either FSIQ or Matrix scores entered as covariates (rehearsal type x group interaction $p = 0.046$ and $p = 0.014$, respectively). There was also a significant main effect of rehearsal type (F(1,22) = 45.16, $p < 0.0001$). The modality x group and rehearsal type x modality interactions both approached significance ($p = 0.10$ and $p = 0.12$, respectively). The articulatory suppression measurement (i.e. no suppression – suppression accuracy) for visual, but not auditory, stimuli was correlated with age ($r = 0.61$, $p = 0.035$) so both lobular damage and age were regressed on patient articulatory suppression measures

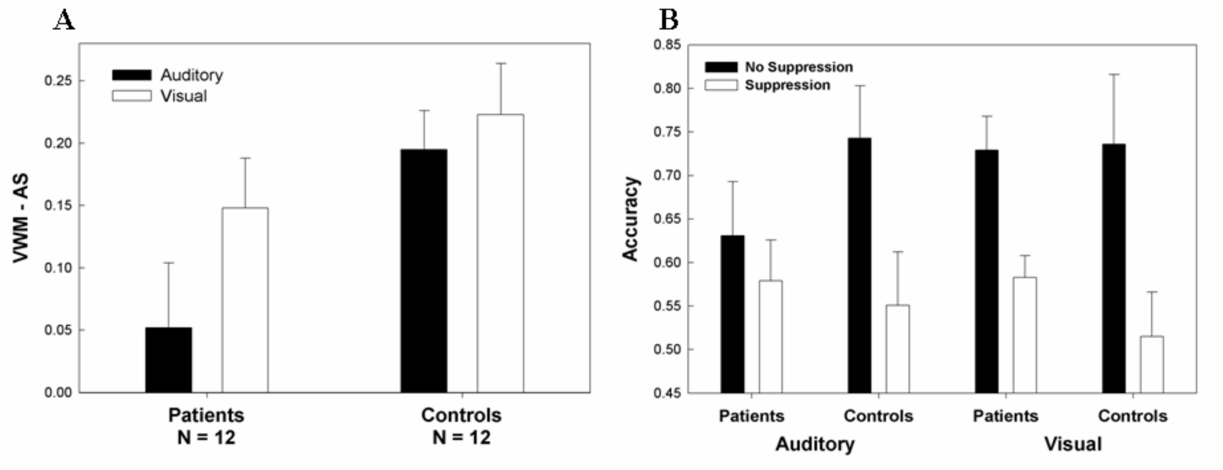

Fig. 4. (A) Articulatory suppression performance (expressed as the difference between non-suppressed and suppressed performance) for patients and controls in auditory and visual modalities. Average accuracy (i.e., proportion of total number of trials answered correctly) for the 12 patients and 12 controls is presented and error bars represent standard error of the mean. (B) Articulatory suppression results with breakdown of non-suppressed and suppressed performance.

for visual stimuli. This analysis indicated that damage to vermian lobule VII (lobule beta t(9) = 3.95, $p$ = 0.0034, multiple R = 0.878, $p$ = 0.0013) and vermian lobule VIII (lobule beta t(9) = 3.97, $p$ = 0.0033, multiple R = 0.879, $p$ = 0.0013) was significantly correlated with impairment in the articulatory suppression measurement for visual stimuli (i.e., as lobule damage increased, the non-suppressed minus suppressed accuracy decreased), with p-values below the Bonferroni threshold. In addition, the following lobules exhibited significant correlations at standard p-value thresholds but did not survive with the Bonferroni correction: left hemispheral lobule IV/V (lobule beta t(9) = 2.54, $p$ = 0.032, multiple R = 0.797, $p$ = 0.011), vermian lobule IV/V (lobule beta t(9) = 2.80, $p$ = 0.0074, multiple R = 0.815, $p$ = 0.0074), vermian lobule VI (lobule beta t(9) = 2.84, $p$ = 0.019, multiple R = 0.818, $p$ = 0.0069), and vermian lobule IX (lobule beta t(9) = 2.82, $p$ = 0.02, multiple R = 0.816, $p$ = 0.0071). In addition the right hemispheral lobule IV/V approached significance (lobule beta t(9) = 1.744, $p$ = 0.11, multiple R = 0.729, $p$ = 0.033). As the auditory articulatory suppression measure was not correlated with age, lobular damage was simply correlated with behavioral measures. In contrast to the visual modality, no lobules were correlated with the auditory articulatory suppression measure.

Analysis of the phonological similarity accuracy measures revealed a main effect of phonological similarity (F(1,22) = 14.92, $p$ = 0.0008), indicating that subjects in general responded with greater accuracy for phonologically dissimilar stimuli (78.9% accuracy) than for similar stimuli (71.7% accuracy). However, there was no significant subject type x phonological similarity interaction or subject type x modality x phonological similarity interaction, indicating that both patients and controls exhibited a standard phonological similarity effect.

Although the ANOVA did not indicate overall group differences in the phonological similarity effect between patients and controls, we noted that in 3 cases for the auditory modality (subjects 01, 02 and 08) and 3 different cases for the visual modality (subjects 03, 06, and 07) behavioral performance was worse than controls by at least 3 standard errors of the mean performance for control subjects. In these cases the performance on the phonologically similar condition was actually slightly better than that for the dissimilar condition. In order to assess anatomical damage that was common to these subjects, a conjunction of the lesions for the 3 subjects in each modality was performed. The results, illustrated in Fig. 5, indicate that damage to hemispheral lobule VIII/VIIB was common to all subjects that showed abnormal performance, and that the modality affected was correlated with the lateralization of the damage such that left cerebellar damage was associated with abnormal performance in the auditory modality and right cerebellar damage was associated with abnormal visual modality performance.

Analysis of free recall data revealed a significant main effect of serial position (F(1,11) = 51.4, $p <$ 0.0001), but the main effect of subject type and the

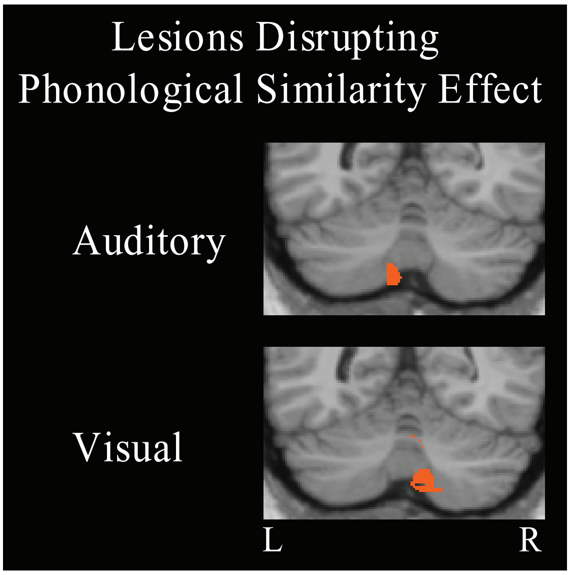

Fig. 5. Conjunction of lesions of subjects exhibiting abnormal phonological similarity effects. The figure represents conjunctions for 3 subjects with abnormal performance for aurally presented stimuli and 3 different subjects who showed abnormal performance for visually presented stimuli. The conjunction region in both cases extends into posterior portions of hemispheral lobules VIII and VIIB.

serial position x subject type interaction were not significant. Figure 6 shows that patients and controls had a similar U-shaped function signifying normal recency effects for both groups. In contrast to the phonological similarity experiment, we did not find individual outliers among the cerebellar patients in serial position performance.

## 4. Discussion

The findings from this study confirm and extend our current knowledge of the role of the cerebellum in cognition and VWM in particular. This is the first systematic study of the neurocognitive sequelae of cerebellar tumor resection in children that focuses on the contributions of individual cerebellar lobules to behavioral impairments. Lobular based analyses enable us to compare these results with activation maps from neuroimaging studies and with predictions from current models of cerebro-cerebellar VWM circuitry.

Our results indicated that cerebellar patients had a significantly reduced memory span for aurally-presented digits relative to control subjects. In contrast to the patient data reported by Silveri and colleagues, memory span did not improve when patients were allowed to point to items rather than speak [60]. While digit span performance for control subjects was superior when digits were presented aurally relative to visual presentation, patients did not exhibit modality differences. These data confirm and extend two previous reports of impaired auditory short-term memory performance in children with cerebellar tumors [13,41]. Although the first of these reports could not distinguish behavioral performance between patients with midline tumors and those with hemispheric lesions [41], a subsequent study by the same researchers concluded that lesions of the midline portions of the cerebellum do not appear to cause cognitive deficits and that short term memory impairments could be explained by hemispheric damage alone [13]. They observed that three of the patients with midline tumors from their initial study who showed memory deficits had lesions which also affected the adjacent cerebellar hemisphere. The lobular analyses from the present study are relevant to these earlier findings and suggest that damage to the left inferior hemispheral lobule VIII, which lies in relatively close proximity to the midline, may be associated with impaired auditory digit span performance. Studying adult cerebellar stroke and tumor patients, Ravizza and colleagues similarly reported impaired digit span performance for aurally-presented stimuli [51]. They further reported greater impairment of digit span for backward relative to forward recall, and no impairment relative to controls for spatial working memory span. Consistent with the present study, they found that digit span performance was correlated with inferior cerebellar damage, although they did not find a laterality effect.

Relative to control subjects, patients' verbal working memory performance in the present study was significantly less affected by articulatory suppression. A lack of an articulatory suppression effect is typically found in patients with articulatory control system (phonological output buffer) deficits [71]. Although the lack of an articulatory suppression effect in the group of cerebellar patients appears to be more pronounced in the auditory modality (Fig. 4B), statistical analyses found significant effects for rehearsal type x group interaction but not for the rehearsal type x modality x group interaction.

Ravizza et al. noted that verbal working memory impairment was correlated with dysarthria severity, consistent with a cerebellar role in articulatory rehearsal, but in contrast to the present study, did not observe significant articulatory suppression effects [51]. Likely sources for the discrepancy between these studies are the ages of the patients and the distributions of the

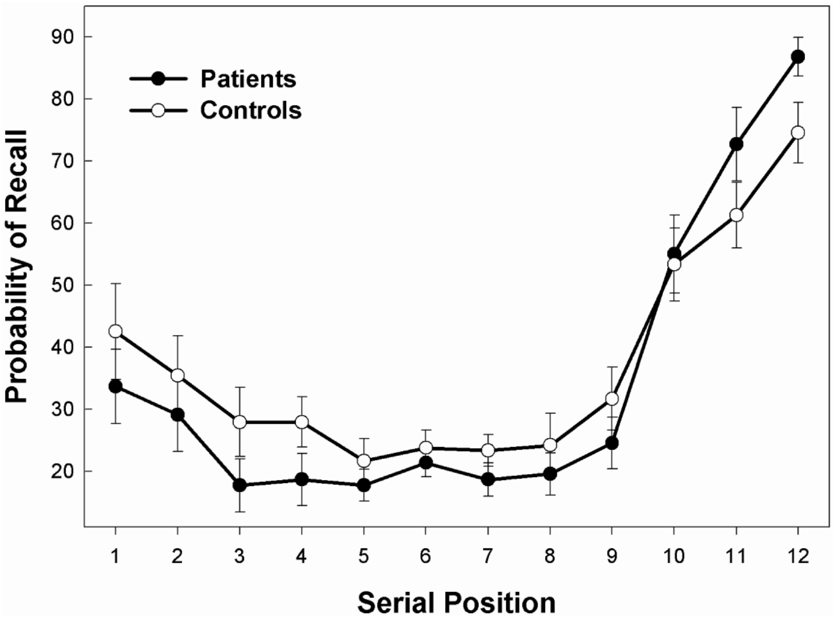

Fig. 6. Free recall performance as a function of serial position of remembered items for patients and controls. Error bars represent standard error of the mean.

lesions. They did note, however, that patients with more superior and anterior cerebellar lesions were more dysarthric and were more affected by the increased demands on rehearsal that occurred from an extended delay period. Our lobular analyses of the articulatory suppression effect indicated that damage to the superior cerebellar hemispheres (lobule IV/V) and large portions of the vermis were particularly correlated with the suppression effect. These results are consistent with the observations of Ravizza et al. as well as other neuroimaging and patient investigations linking superior cerebellum to articulation [2,3,59,70]. Event-related neuroimaging investigations have found that superior cerebellar activations tend to be strongly related to the encoding phase of the verbal working memory task [14, 15] and verbal working memory performance can be impaired by transcranial magnetic stimulation administered just after the encoding period [22], suggesting that the superior cerebellar role in articulation may be to assist in orthographic to phonological conversion of visual information and/or to set up an initial motor articulatory trajectory.

Statistical analyses of patients and control subjects in the present investigation revealed overall normal phonological similarity and recency effects. However, inspection of individual patient performance did reveal outlier performance in the phonological similarity experiment for 3 patients in the auditory modality and 3 patients in the visual modality. Conjunction of the lesion data for these subjects suggested that left hemispheral lobule VIII damage was associated with an abnormal phonological similarity effect with aurally presented stimuli, while damage to the same region on the right side was associated with an abnormal phonological similarity effect in the visual modality. These results are consistent with the findings of Justus and colleagues who found that patients with lesions which included the inferior cerebellum, either unilaterally or bilaterally, disrupted the phonological store and yielded a negative phonological similarity effect [33]. Similarly, Chiricozzi et al. [17] reported a case study of a patient with cerebellar damage to left hemispheral lobule VIII and right hemispheral lobule V that showed impaired phonological similarity effects as well as other signs of impaired phonological storage. It can be further noted that our group results may have underestimated disruption of phonological similarity effects by using a recognition rather than recall procedure. Similarly, non-disruption of the recency effect in patients was observed using visually presented letters, whereas impairment might have been more readily detected with auditory presentation.

With respect to the concordance of the present results with previous findings from functional neuroimaging, we have consistently found with visually presented letters in a Sternberg verbal working memory task that right HVIII/HVIIB is activated mostly during the maintenance phase of the memory task, but not during motoric rehearsal conditions in which phonological storage is not required. On the basis of this observation as well as evidence from neuroanatomy regarding cerebro-cerebellar connectivity we have suggested that this inferior cerebellar region is linked with temporal/parietal neocortical regions that have been implicated in phonological storage [15,16,23]. As described below, the emphasis from neuroimaging on right inferior cerebellum may have been biased from the use of visually-presented letters. Recent data from our lab indicates that with aurally-presented letters, activation of left inferior cerebellar regions (as well as right) is much more apparent [36]. The presence of overall phonological similarity and recency effects in the present study (albeit a null result) are not inconsistent with the neuroimaging data because lobular damage to HVIII and HVIIB was small in most patients. However, the disruptive effect of inferior cerebellar damage on the phonological similarity effect in a subset of our patients is consistent with previous neuroimaging findings.

Functional neuroimaging has also revealed activation in superior cerebellar hemispheres (right HVI, and left Crus I) as well as in the vermis (VI and VII) when contrasting high vs. low load conditions for both working memory and motoric rehearsal (non-memory) tasks, suggesting that these regions might be involved in common articulatory or encoding operations for both types of tasks [16,23]. We found that patients were significantly less disrupted by articulatory suppression than controls, indicating a possible deficit in articulatory control. Although damage to right HVI and left Crus I was also very modest in our patients (mean of 9.7 and 6.2 percent, respectively), damage to the vermis was extensive and the significant correlation of damage to vermian lobule VI and VII with articulatory suppression measures is consistent with the functional neuroimaging evidence.

In summary, damage to left hemispheral lobule VIII in the present study was associated with reduced digit span to auditory stimuli. There is some indication from our data that damage to this lobule may affect phonological storage. The abnormal articulatory suppression effects as well as the selective disruption of auditory digit span are patterns similar to those observed in patients with lesions in premotor, Rolandic, and insular regions who are diagnosed with primary impairment in the articulatory control system [71]. While these similarities suggest that cerebellar lesions in the present study caused impairment of the articulatory control system, two observations should be considered. The first is that no improvement in auditory digit span was observed when subjects were allowed to point rather than to respond verbally. If articulatory control was the primary deficit for the cerebellar patients, one would expect that bypassing the articulatory system through pointing would have improved performance. A memory advantage from the pointing procedure has been viewed as evidence of an impaired articulatory control system, and such an advantage from pointing has been observed in a case study of verbal working memory impairment in a cerebellar patient [60]. The second observation is that the patients showing the most severe visual articulatory control deficits were not the same patients that showed the most disruption of the phonological similarity effect. In patients with articulatory control deficits due to left frontal lesions, phonological similarity effects are typically preserved for the auditory modality, but impaired for visual stimuli, due to the need for the articulatory system in translating the visual letters into a phonological code [71]. We also note that while damage to lobule HVIII was highly correlated with reduced auditory digit span performance, this lobule was not correlated with behavioral suppression effects – rather, such effects were correlated with superior cerebellar and vermis damage. Although the articulatory suppression data suggests impairment of the articulatory control system, from the overall pattern of the data we hypothesize that damage to left lobule HVIII may interfere with normal encoding of auditory stimuli into phonological storage, possibly affecting item sequencing [1,42,44] or item associations [38] that may normally contribute to phonological loop execution. Such an effect would be generally consistent with a hypothesized role of the cerebellum in sensory acquisition [10,49]. In support of this hypothesis, functional MRI observations from our laboratory, directly contrasting activation in a Sternberg verbal working memory task under conditions of aurally vs. visually presented stimuli, have shown that while right lateral HVIII (HVIIIA) and HVIIB is activated for both auditory and visual presentation of letters, only the auditory condition activates more medial inferior cerebellar regions including bilateral medial HVIII (HVIIIB) [36, 37]. The concordance of these functional neuroimaging data, in which the modality of the letters presented was the only difference in conditions, with the results of the present study suggest that the medial inferior hemispheres may be a critical entry point for auditory information in the cerebellum, and that damage to this region can impair working memory performance in the auditory modality.

## Acknowledgements

This study was supported by the Lucile Packard Foundation for Children's Health, NIH (MH060234) and the Stanford Medical Scientist Training Program. We thank Ruth Rosenblum and Meghan Denzel for assistance with subject recruitment.
